# GV-971 Ameliorates Chronic Restraint Stress-Induced Depression-like Phenotypes Accompanied by Reshaping of the Microbiota–Gut–Brain Axis

**DOI:** 10.3390/md24060189

**Published:** 2026-05-24

**Authors:** Zhuandi He, Yali Nie, Changcai Li, Guangqiang Sun, Wei Zheng, Hongchun Liu, Meiyu Geng, Jingwei Tian, Yu Zhang

**Affiliations:** 1 School of Pharmacy, Key Laboratory of Molecular Pharmacology and Drug Evaluation (Yantai University), Ministry of Education, Collaborative Innovation Center of Advanced Drug Delivery System and Biotech Drugs in Universities of Shandong, Yantai University, Yantai 264005, China; 2 Shandong Laboratory of Yantai Drug Discovery, Bohai Rim Advanced Research Institute for Drug Discovery, Yantai 264117, China; 3 State Key Laboratory of Drug Research, Shanghai Institute of Materia Medica, Chinese Academy of Sciences, Shanghai 201203, China

**Keywords:** GV-971, depression, microbiota–gut–brain axis, tryptophan metabolism, neuroinflammation, fecal microbiota transplantation

## Abstract

Depression is increasingly linked to microbiota–gut–brain axis dysfunction, yet current monoaminergic antidepressants show limited efficacy. This study investigated the therapeutic potential and underlying mechanisms of GV-971, a marine-derived oligosaccharide, in a chronic restraint stress (CRS) mouse model. We first established that 8 h of daily restraint for 4–8 weeks induces a stable depression-like phenotype characterized by behavioral despair and significant reduction in peripheral monoamine neurotransmitters (5-HT and norepinephrine). GV-971 treatment robustly attenuated CRS-induced depression- and anxiety-like behaviors, restored hippocampal serotonin levels, reduced elevated plasma corticosterone concentrations, and ameliorated CRS-induced adrenal cortical hyperplasia. Mechanistically, GV-971 significantly suppressed neuroinflammation by inhibiting microglial hyperactivation in the prefrontal cortex and hippocampus. Concurrently, it repaired intestinal barrier dysfunction, evidenced by reduced permeability, restored mucosal integrity, and recovered goblet cell numbers. Crucially, integrated shot-gun metagenomics and plasma metabolomics revealed that GV-971 not only reshaped microbial taxonomy but also functionally recalibrated the gut ecosystem. It enriched beneficial taxa (e.g., *Bifidobacterium pseudolongum*, *Bacteroides uniformis*) and specific metabolic pathways, leading to increased short-chain fatty acids (valeric and caproic acids) and a significant reduction in plasma levels of tryptophan–kynurenine pathway metabolites, specifically the neurotoxic compounds kynurenine and quinolinic acid. Fecal microbiota transplantation (FMT) from GV-971-treated donors partially recapitulated the antidepressant and gut-protective effects in CRS recipients, confirming a causal role for the remodeled microbiota. Collectively, GV-971 exerts antidepressant effects by coordinately remodeling the gut microbiota, normalizing tryptophan and SCFA metabolism, restoring gut barrier integrity, and dampening central neuroinflammation, supporting its potential as a novel gut–brain axis-targeted therapy for depression.

## 1. Introduction

Major depressive disorder (MDD) is a disabling psychiatric condition characterized by persistent low mood and marked impairment in social and occupational functioning [1]. With an estimated 322 million affected individuals globally, the disease burden falls disproportionately on older adults and women, who have an approximately 1.5-fold-higher risk than men [2]. Although monoamine-modulating agents, particularly selective serotonin reuptake inhibitors (SSRIs), remain the cornerstone of first-line pharmacotherapy in clinical practice, their utility is often hampered by a lag in therapeutic onset and significant adverse gastrointestinal effects [3]. Recent guideline revisions underscore the limitations of current standards, revealing that almost half of patients do not respond sufficiently to first-line agents, while nearly 70% of patients with MDD experience residual symptoms. These may include anxiety, impaired cognition, fatigue and sleep disturbance [4]. The enduring discrepancy between the therapeutic ceiling of conventional monoaminergic agents and the unmet needs of clinical practice underscores the imperative for next-generation interventions that target novel biological pathways while ensuring superior tolerability.

The biological underpinnings of depression extend well beyond monoamine depletion, encompassing hyperactivation of the hypothalamic–pituitary–adrenal (HPA) axis, deficits in neurotrophic support, mitochondrial dysfunction and sustained neuroinflammation [5]. Disturbances of the microbiota–gut–brain axis are increasingly regarded as a key node that integrates these processes and shapes disease progression [6]. Data from preclinical and clinical studies, particularly fecal microbiota transplantation (FMT) experiments, suggest that gut dysbiosis does more than simply accompany mood pathology; in many contexts, it acts as a principal trigger for depression-like phenotypes [7,8]. This bidirectional circuitry relies heavily on microbial control of metabolic pathways, notably those involving tryptophan and short-chain fatty acids (SCFAs). When microbial homeostasis is disrupted, the breakdown of the intestinal barrier and the loss of protective indole derivatives bias tryptophan catabolism toward pro-inflammatory and neurotoxic routes—a phenomenon often referred to as metabolic “shunting” [9,10]. The resulting peripheral inflammatory and metabolic signals cross the blood–brain barrier, drive aberrant microglial activation and sustain central neuroinflammatory cascades, thereby maintaining the depressive state [11]. These observations have prompted efforts to develop antidepressant strategies that explicitly target and recalibrate the “gut microbiota–metabolism–neuroimmune” network [12,13].

GV-971 (sodium oligomannate) is a marine-derived, low-molecular-weight acidic oligosaccharide approved for clinical use in neurodegenerative disorders and is regarded as well tolerated, with a defined capacity to act on the microbiota–gut–brain axis. Available evidence indicates that GV-971 markedly reshapes gut microbial communities and limits the aberrant accumulation of pathogenic amino acid-related metabolites [14,15,16]. Through these microbiota-dependent effects, GV-971 dampens peripheral and central inflammatory responses while increasing serotonin (5-HT) availability in the intestinal tract and systemic circulation. Such combined properties—microbial remodeling, neuroinflammatory suppression and enhancement of serotonergic tone—closely mirror core pathological cascades implicated in major depression and have led to the hypothesis that GV-971 could act as a microbiota-directed antidepressant. It remains unclear, however, whether this agent can alleviate depressive phenotypes by correcting defined “microbiota–metabolome” disturbances and, in turn, limiting microglial hyperactivation. To address this, the present study employs a chronic restraint stress (CRS) mouse model to evaluate the antidepressant-like efficacy of GV-971 and to delineate the gut–brain signaling mechanisms that may underlie its therapeutic actions.

## 2. Results

### 2.1. Eight Hours per Day of Chronic Restraint for 4–8 Weeks Induces a Stable Depression-like Phenotype in Mice

**Figure 1:**
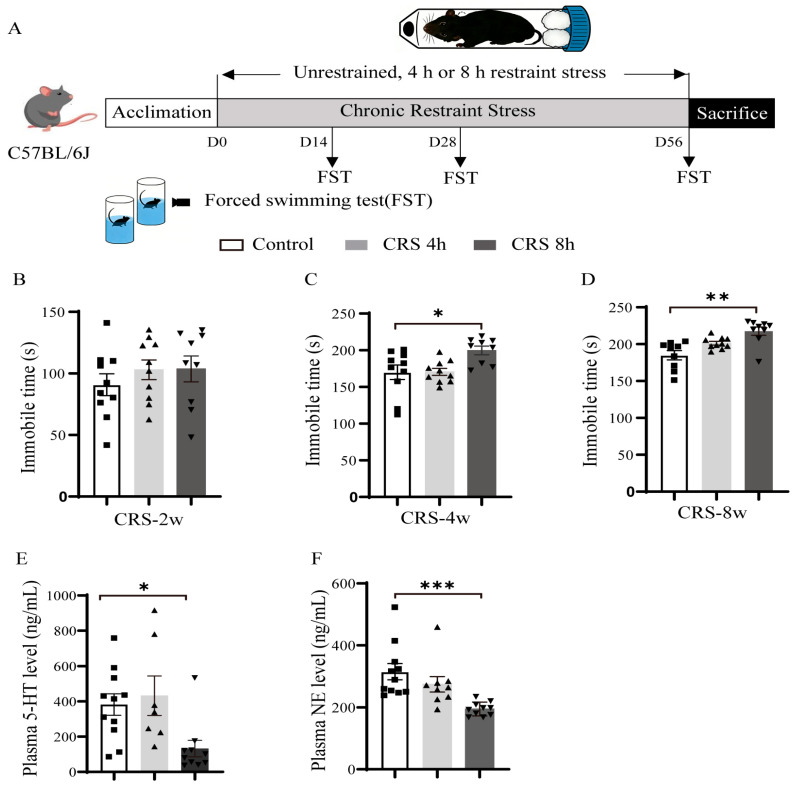
Eight hours per day of chronic restraint for 4–8 weeks induces a stable depression-like phenotype in mice. (**A**) Schematic of the experimental design. (**B**) Immobility time in the forced swim test (FST) after 2 weeks of chronic restraint stress (CRS). (**C**) Immobility time in the FST after 4 weeks of CRS. (**D**) Immobility time in the FST after 8 weeks of CRS. (**E**) Plasma 5-hydroxytryptamine (5-HT) levels. (**F**) Plasma norepinephrine (NE) levels. Data are presented as mean ± SEM, *n* = 7–11 mice per group. Control group (represented by open squares and black squares), CRS 4h group (light gray filled square and black upward triangles), CRS 8h group (represented by dark gray filled squares and black downward triangles). Statistical significance was determined by one-way ANOVA with Tukey’s post hoc test. Non-normally distributed data were analyzed using the Kruskal–Wallis test with Bonferroni’s correction. * *p* < 0.05, ** *p* < 0.01, and *** *p* < 0.001 compared to the control group.

To establish a robust and objective chronic restraint stress (CRS) model of depression, the restraint parameters (daily duration and total exposure) were systematically optimized. Immobility time in the forced swim test (FST) was used as the primary behavioral endpoint. CRS-induced depression-like behavior showed clear time- and intensity-dependent effects. After 2 weeks of CRS, both the 4 h and 8 h restraint groups exhibited a trend toward increased immobility relative to the naïve control group, but these changes did not reach statistical significance and overt depression-like behavior was not evident (Figure 1B). By 4 weeks, immobility time in the 8 h restraint group was significantly prolonged compared with controls (from 169.95 ± 9.85 s to 199.77 ± 5.86 s, *p* = 0.021; Figure 1C). When the CRS period was extended to 8 weeks, immobility time in the 4 h group showed an increasing trend, and in the 8 h group, it significantly increased from 184.93 ± 6.32 s to 217.64 ± 5.66 s (*p* = 0.001), indicating a typical depression-like despair phenotype (Figure 1D). Peripheral neurochemical measurements further supported an intensity-dependent effect of restraint stress. At week 8, mice in the 8 h group displayed marked depletion of plasma monoamine neurotransmitters. Compared with controls, plasma 5-HT levels decreased from 381.92 ± 61.02 ng/mL to 132.02 ± 46.77 ng/mL (*p* = 0.012; Figure 1E), and NE levels fell from 315.46 ± 26.29 ng/mL to 194.84 ± 7.01 ng/mL (*p* < 0.001; Figure 1F). In contrast, 4 h restraint did not significantly alter plasma 5-HT or NE, suggesting that 8 h of daily restraint is more effective at inducing peripheral neuroendocrine disruption. Overall, these behavioral and neurochemical data suggest that 4 h/d CRS exerts only a mild impact on the monoamine system, whereas 8 h/d for 4–8 weeks induces both emerging behavioral despair and progressive monoamine dysregulation. Under the 8 h/d regimen, mice developed a stable depression-like phenotype that was already apparent at week 4 and remained sustained through week 8.

### 2.2. GV-971 Attenuates CRS-Induced Behavioral Abnormalities in Mice

**Figure 2:**
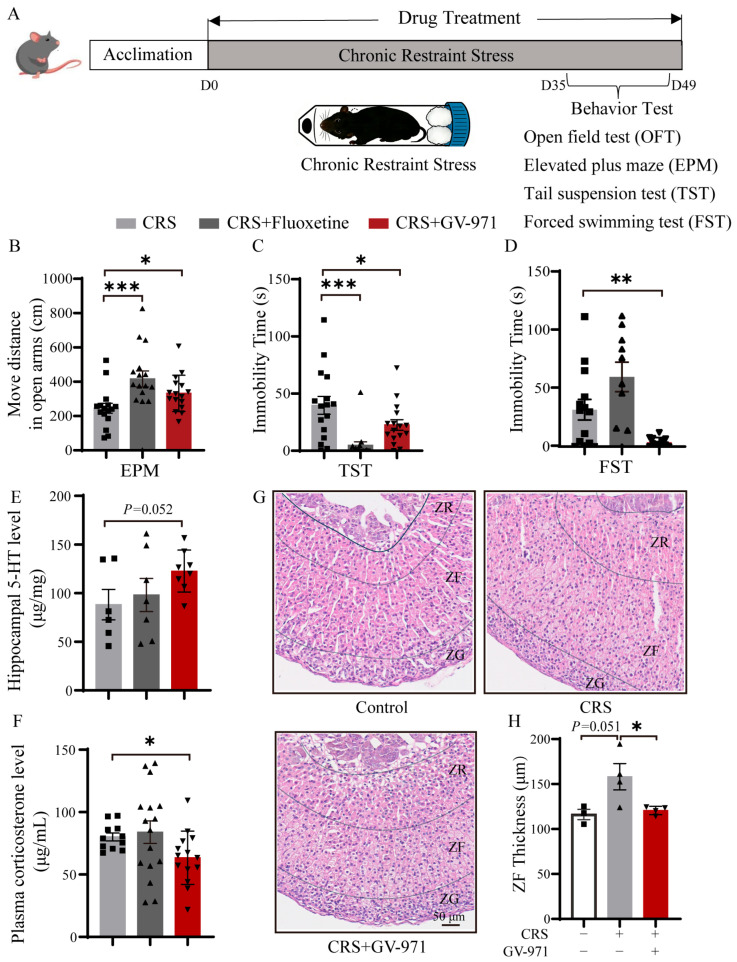
GV-971 ameliorates CRS-induced depression-like phenotypes in mice. (**A**) Schematic illustration of the CRS procedure and drug administration timeline. (**B**) Distance traveled in the open arms of the elevated plus maze (EPM), *n =* 16 mice per group. (**C**,**D**) Immobility time in the tail suspension test (TST; **C**), *n =* 16 mice per group, and in the forced swim test (FST; **D**), *n =* 10–14 mice per group. (**E**) Hippocampal 5-hydroxytryptamine (5-HT) levels, *n =* 6–8 mice per group. (**F**) Plasma corticosterone (CORT) levels, *n =* 11–16 mice per group. (**G**) Representative H&E-stained sections of adrenal gland from each group (scale bar = 50 μm). CRS group (represented by light gray filled squares and black squares), CRS+Fluoxetine group (represented by dark gray filled squares and black upward triangles), and CRS+GV-971 group (represented by red filled squares and black downward triangles). (**H**) Quantification of the thickness of the adrenal zona fasciculata (ZF), *n =* 3–4 mice per group. Fluoxetine was employed as a positive control. Data are presented as mean ± SEM. Statistical significance for comparisons between the CRS group and treatment group was determined using the independent-samples *t*-test. * *p* < 0.05, ** *p* < 0.01, and *** *p* < 0.001 compared to the CRS group.

To determine whether GV-971 could rescue the behavioral alterations associated with chronic restraint stress (CRS), mice were assessed via the elevated plus maze (EPM), tail suspension test (TST), and forced swim test (FST) during weeks 5–7 of CRS, with fluoxetine serving as the positive control. In the EPM, both GV-971 and fluoxetine significantly increased open-arm distance relative to CRS mice (Figure 2B). Values increased from 245.38 ± 29.10 cm in the CRS group to 332.18 ± 26.49 cm in the GV-971 group (*p* = 0.035) and 444.09 ± 46.50 cm in the fluoxetine group (*p* < 0.001), consistent with an anxiolytic-like effect. In the TST, immobility time was markedly reduced by both drugs (Figure 2C), from 39.71 ± 7.81 s in CRS mice to 19.24 ± 3.42 s after GV-971 treatment (*p* = 0.026) and to 1.56 ± 0.59 s after fluoxetine (*p* < 0.001). In the FST, GV-971 again significantly shortened immobility time (Figure 2D), from 31.61 ± 8.85 s in CRS mice to 3.85 ± 1.14 s (*p* = 0.008), whereas fluoxetine produced a numerically higher immobility time (59.71 ± 12.66 s) that did not differ significantly from the CRS group.

In biochemical analyses, hippocampal 5-HT levels tended to be higher in GV-971-treated mice than in CRS controls (Figure 2E), rising from 88.21 ± 15.67 ng/mg to 122.81 ± 7.62 ng/mg (*p* = 0.052), whereas the fluoxetine group showed 5-HT levels of 98.22 ± 17.11 ng/mg, which were not significantly different from those of the CRS group. Plasma CORT measurements demonstrated that GV-971 significantly lowered CORT concentrations from 80.14 ± 3.14 ng/mL to 63.45 ± 5.71 ng/mL (*p* = 0.027) (Figure 2F). By contrast, fluoxetine-treated mice displayed CORT levels of 83.91 ± 9.03 ng/mL, with no significant improvement relative to CRS controls. Adrenal histology further supported a normalizing effect of GV-971 on the stress axis. H&E staining revealed evident adrenal cortical hyperplasia and thickening in CRS mice, whereas GV-971-treated animals showed a cortical morphology closer to that of control mice (Figure 2G). Morphometric analysis confirmed a trend towards increased cortical thickness in the CRS group compared with controls (116.13 ± 5.78 μm in the control group vs. 158.14 ± 14.53 μm in the CRS group, *p* = 0.051). GV-971 significantly reduced cortical thickness to 120.54 ± 2.33 μm (*p* = 0.044 vs. CRS), suggesting that GV-971 suppresses CRS-induced adrenal cortical hypertrophy and partially restores normal adrenal structure (Figure 2H). Collectively, these data indicate that GV-971 not only rescues CRS-induced behavioral deficits but also uniquely targets the underlying neuroendocrine dysregulation. Unlike fluoxetine, which showed limited efficacy on the HPA axis in this model, GV-971 demonstrated a comprehensive therapeutic profile by concurrently alleviating depression-like behaviors, trending toward restoring central serotonin, and significantly normalizing peripheral stress markers and adrenal morphology.

### 2.3. GV-971 Inhibits Abnormal Microglial Activation in CRS Mice

**Figure 3:**
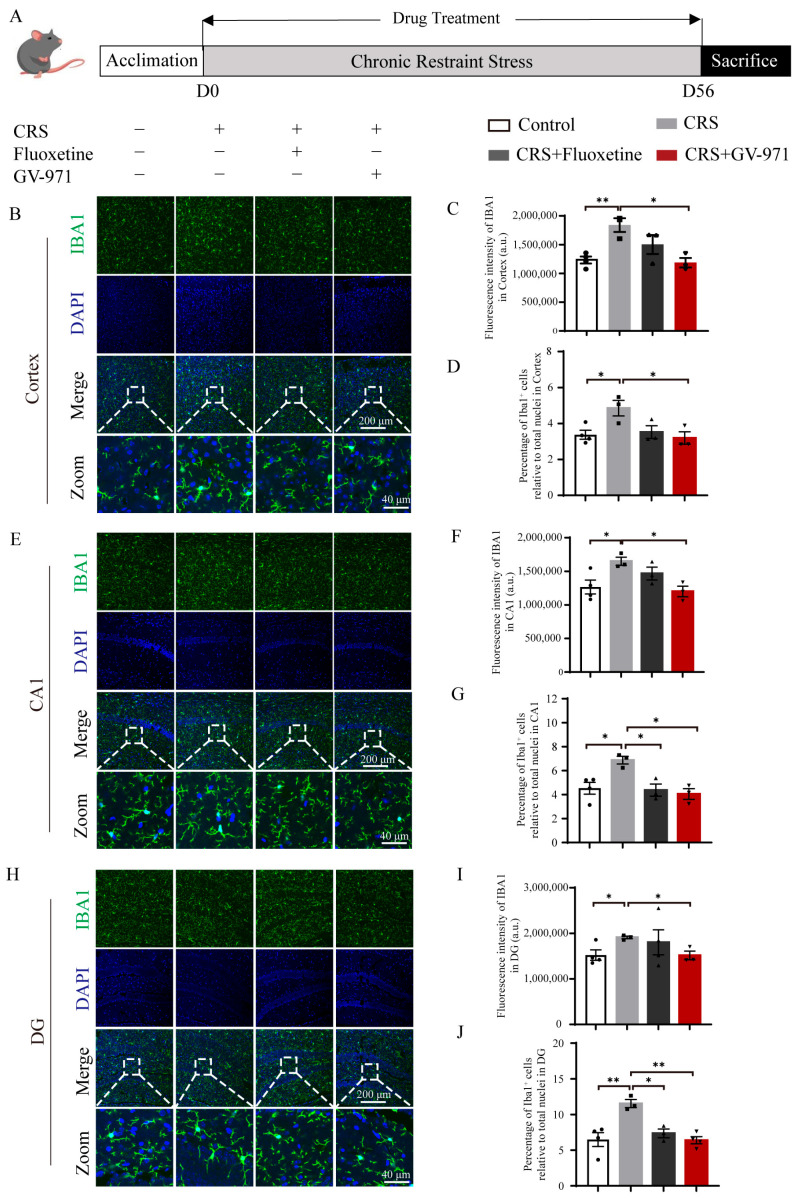
GV-971 inhibits abnormal activation of microglia in the brains of CRS model mice. (**A**) Schematic diagram of the experimental design, including CRS modeling and drug administration. (**B**–**D**) Representative IBA1 immunofluorescence images (**B**), quantification of IBA1 fluorescence intensity (**C**), and analysis of the percentage of Iba1^+^ cells relative to total nuclei (**D**) in the cerebral cortex. (**E**–**G**) Representative IBA1 immunofluorescence images (**E**), quantification of IBA1 fluorescence intensity (**F**), and analysis of the percentage of Iba1^+^ cells relative to total nuclei (**G**) in the hippocampal CA1 region. (**H**–**J**) Representative IBA1 immunofluorescence images (**H**), quantification of IBA1 fluorescence intensity (**I**), and analysis of the percentage of Iba1^+^ cells relative to total nuclei (**J**) in the hippocampal DG region. Control group (represented by open squares and black circles), CRS group (represented by light gray filled squares and black squares), CRS+Fluoxetine group (represented by dark gray filled squares and black upward triangles), and CRS+GV-971 group (represented by red filled squares and black downward triangles). Fluoxetine was employed as a positive control. Data are presented as mean ± SEM, *n =* 3–4 mice per group. Statistical significance for comparisons between the CRS group and treatment group was determined using the independent-samples *t*-test. * *p* < 0.05 and ** *p* < 0.01 compared to the CRS group. Scale bars: 200 μm for main images and 40 μm for zoom images.

To investigate the effect of GV-971 on chronic restraint stress (CRS)-induced neuroinflammation, mice were exposed to CRS for 56 days and received GV-971 or fluoxetine (positive control) according to the schedule shown in Figure 3A. At the end of the experiment, brain tissue was collected, and immunofluorescence staining for the microglial marker IBA1 was performed in the cortex, hippocampal CA1 region and dentate gyrus (DG). Morphological analysis (Figure 3B,E,H) showed that in control mice, IBA1-positive microglia (green) were sparse in the cortex and hippocampus (CA1 and DG) and exhibited thin, highly ramified processes, consistent with a resting state. In contrast, CRS markedly increased the number of IBA1-positive microglia in these regions, with stronger fluorescence signals and a characteristic activated morphology, including enlarged cell bodies and retracted processes. Treatment with GV-971 markedly attenuated this abnormal microglial activation, as indicated by a reduction in the number of IBA1-positive cells and a gradual restoration of microglial morphology towards the resting phenotype observed in controls. Quantification of IBA1 fluorescence intensity in the cortex, hippocampal CA1 and DG is shown in Figure 3C,F,I, whereas the percentage of Iba1^+^ cells relative to total nuclei is quantified in Figure 3D,G,J. In the cortex, IBA1 fluorescence intensity was significantly increased in CRS mice compared with control mice, rising from 1,249,710 ± 58,939 a.u. to 1,839,068 ± 119,058 a.u. (*p* = 0.0047). GV-971 treatment significantly reduced this increase to 1,185,823 ± 82,970 a.u. (*p* = 0.011 vs. CRS). Concurrently, the percentage of Iba1^+^ cells was significantly increased in CRS mice compared with control mice, rising from 3.37 ± 0.25% to 4.85 ± 0.44% (*p* = 0.047). Both fluoxetine and GV-971 treatments reduced this increase: fluoxetine lowered it to 3.52 ± 0.36% (*p* = 0.099 vs. CRS), and GV-971 reduced it to 3.19 ± 0.35% (*p* = 0.037 vs. CRS), with no significant differences observed between the treatment group and the control group. No significant differences in IBA1 fluorescence intensity were observed in the fluoxetine group compared with controls.

In the hippocampal CA1 region, IBA1 fluorescence intensity similarly increased from 1,266,011 ± 104,043 a.u. in controls to 1,648,689 ± 60,566 a.u. in CRS mice (*p* = 0.035), and was significantly reduced to 1,199,010 ± 79,509 a.u. by GV-971 treatment (*p* = 0.011 vs. CRS). The percentage of Iba1^+^ cells similarly increased from 4.53 ± 0.49% in controls to 6.87 ± 0.32% in CRS mice (*p* = 0.022). Fluoxetine treatment significantly reduced this elevation to 4.38 ± 0.51% (*p* = 0.022 vs. CRS), while GV-971 treatment decreased it to 4.05 ± 0.44% (*p* = 0.011 vs. CRS), both restoring levels comparable to those of the control group.

In the hippocampal DG, IBA1 fluorescence intensity was also significantly elevated by CRS, increasing from 1,521,988 ± 114,351 a.u. in controls to 1,909,210 ± 28,694 a.u. (*p* = 0.037), whereas GV-971 significantly lowered it to 1,513,386 ± 96,441 a.u. (*p* = 0.017 vs. CRS). No significant differences were observed in the fluoxetine group in the cortex, CA1 or DG. The percentage of Iba1^+^ cells was also markedly elevated by CRS, increasing from 6.48 ± 0.98% in controls to 11.54 ± 0.57% (*p* = 0.0031). Fluoxetine treatment significantly lowered it to 7.36 ± 0.50% (*p* = 0.017 vs. CRS), and GV-971 treatment reduced it to 6.39 ± 0.50% (*p* = 0.0028 vs. CRS), with GV-971 restoring levels nearly identical to those of the control group.

These morphological and quantitative data collectively indicate that GV-971 effectively attenuates CRS-induced microglial hyperactivation in the cortex and hippocampus, suggesting mitigated neuroinflammatory responses.

### 2.4. GV-971 Ameliorates CRS-Induced Intestinal Barrier Dysfunction and Colonic Pathology

**Figure 4:**
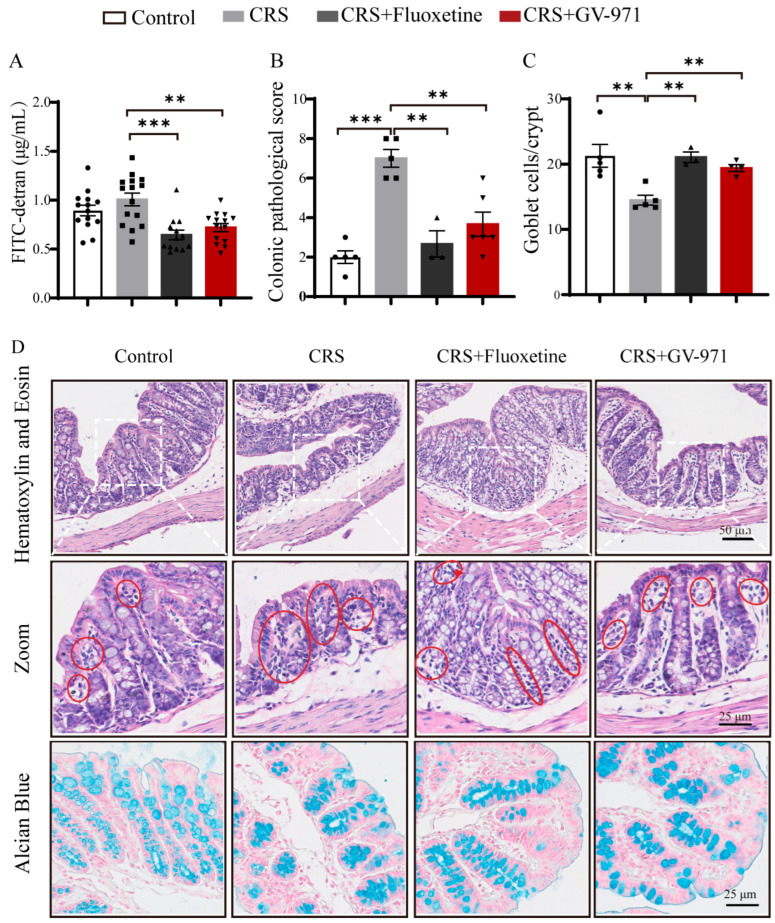
GV-971 attenuates CRS-induced intestinal barrier disruption and colonic inflammation. (**A**) Intestinal permeability was assessed by measuring serum FITC-dextran levels, *n =* 13–15 mice per group. (**B**) Histological injury scores were used to evaluate the degree of colonic tissue damage. (**C**) Quantification of goblet cells per colonic crypt. (**D**) Representative images of H&E and Alcian blue staining in colonic tissue. For (**B**–**D**), *n =* 3–6 mice per group. Red circles indicate leukocyte infiltrationa. Fluoxetine was employed as a positive control. Control group (represented by open squares and black circles), CRS group (represented by light gray filled squares and black squares), CRS+Fluoxetine group (represented by dark gray filled squares and black upward triangles), and CRS+GV-971 group (represented by red filled squares and black downward triangles).Data are presented as mean ± SEM. Statistical significance for comparisons between the CRS group and treatment group was determined using the independent-samples *t*-test. ** *p* < 0.01 and *** *p* < 0.001 relative to the CRS group. Scale bars: 50 μm for H&E main images and 25 μm for H&E zoom images and Alcian blue images.

Beyond its central effects, the therapeutic efficacy of GV-971 likely stems from its ability to rectify upstream disturbances at the gut level. Therefore, we examined whether the drug could reverse CRS--induced gut barrier dysfunction and mucosal damage. Intestinal barrier function was assessed using the FITC-dextran permeability assay (Figure 4A). Serum FITC-dextran reached 1.01 ± 0.07 μg/mL in CRS mice, whereas control mice showed levels of 0.89 ± 0.05 μg/mL, suggesting a slight trend toward increased intestinal permeability. Both GV-971 and fluoxetine markedly reduced serum FITC-dextran relative to the CRS group. In the GV-971 group, serum FITC-dextran decreased to 0.72 ± 0.04 μg/mL (*p* = 0.0011 vs. CRS), and in the fluoxetine group it decreased to 0.64 ± 0.05 μg/mL (*p* < 0.001 vs. CRS), indicating restoration of barrier function. Colonic pathology was then evaluated by H&E staining, Alcian blue staining, and histological injury scoring. In control mice, the colonic mucosa was intact, epithelial cells were regularly arranged, and goblet cell morphology was preserved. By contrast, CRS caused pronounced mucosal injury, characterized by disruption of glandular and cellular architecture, crypt shortening, a moderate reduction in goblet cells, diffuse leukocyte infiltration in the lamina propria, and prominent inflammatory cell accumulation in the submucosa (Figure 4D). These alterations were accompanied by a marked increase in the histological injury score, which rose from 2.00 ± 0.32 in control mice to 7.00 ± 0.45 in CRS mice (*p* < 0.001; Figure 4B). Both treatments ameliorated these pathological abnormalities, with improved structural preservation and reduced inflammatory infiltration. Consistent with these observations, the histological injury score fell to 3.67 ± 0.62 in the GV-971 group (*p* = 0.0022) and to 2.67 ± 0.67 in the fluoxetine group (*p* = 0.0013), as compared with untreated CRS mice. Alcian blue staining provided additional evidence of mucus barrier disruption in CRS mice (Figure 4D). Control colons displayed dense, regularly arranged goblet cells along the mucosal surface, whereas CRS colons showed marked goblet cell depletion with a sparse and irregular distribution pattern, consistent with severe compromise of the mucus-secreting barrier. Quantitative counting of goblet cells per crypt corroborated these histological observations (Figure 4C). Goblet cell numbers declined from 21.28 ± 1.75 per crypt in control mice to 14.48 ± 0.76 per crypt in CRS mice (*p* = 0.0073). Following treatment, goblet cell counts were partially restored: GV-971 increased the number of goblet cells to 19.64 ± 0.54 per crypt (*p* = 0.0015 vs. CRS), while fluoxetine raised it to 21.07 ± 0.79 per crypt (*p* = 0.0013 vs. CRS), nearly matching control levels. Together with the FITC-dextran permeability data and histological injury scoring, these results show that GV-971 not only mitigates CRS-induced colonic pathology but also markedly facilitates functional recovery of the intestinal mucus barrier.

### 2.5. GV-971 Remodels the Gut Microbiota in a Mouse Model of CRS-Induced Depression

**Figure 5:**
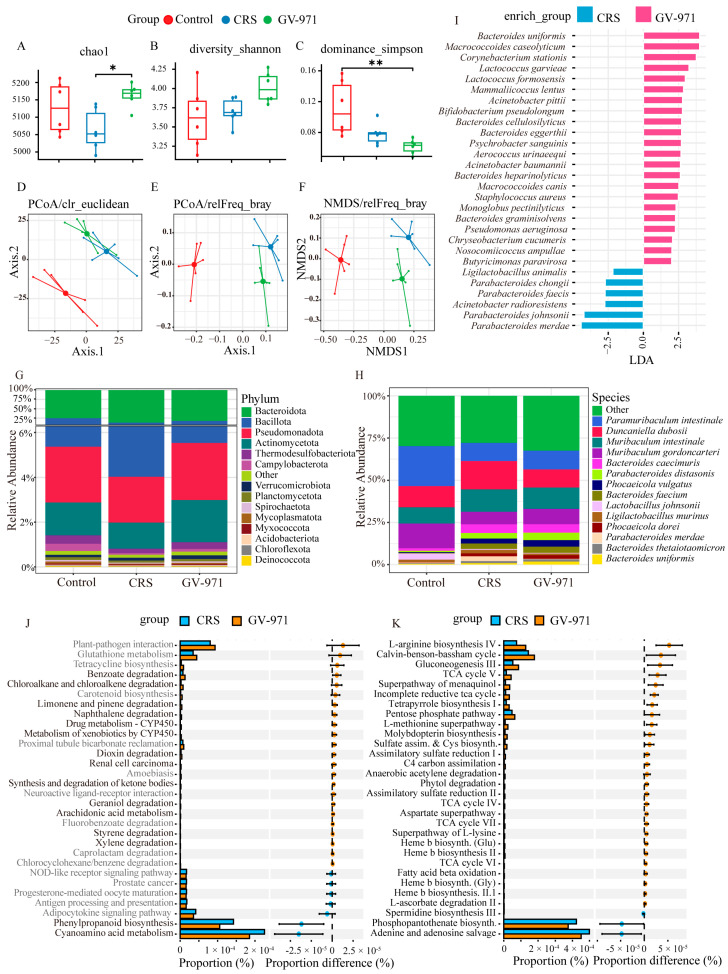
GV-971 reshapes the gut microbiome in mice subjected to chronic restraint stress. Fecal samples were collected after 8 weeks of CRS and analyzed by metagenomic sequencing. GV-971 attenuated CRS-associated gut microbiota dysbiosis by modifying microbial diversity, taxonomic composition, and functional potential. (**A**–**C**) α-diversity analyses: boxplots of the Chao1 richness index (**A**), Shannon diversity index (**B**), and Simpson dominance index (**C**). (**D**–**F**) β-diversity analyses: (**D**) principal coordinate analysis (PCoA) based on CLR-transformed Euclidean distance; (**E**) PCoA based on Bray–Curtis distance calculated from relative abundance; (**F**) non-metric multidimensional scaling (NMDS) based on Bray–Curtis distance calculated from relative abundance. (**G**,**H**) Taxonomic composition of the gut microbiota: stacked bar plots showing the relative abundance of gut microbiota at the phylum level (**G**) and at the species level (**H**). (**I**) LEfSe analysis showing linear discriminant analysis (LDA) scores of differential bacterial taxa between the CRS and GV-971 groups. (**J**,**K**) Functional profiling of gut microbial communities: (**J**) bar plot of differentially enriched KEGG pathways; and (**K**) MetaCyc-based analysis of differential metabolic pathways. *n* = 6 mice per group. Differentially abundant taxonomic biomarkers were identified using LEfSe (microbi-ome Marker package; LDA score > 2, * *p* < 0.05 and ** *p* < 0.01).

Given that gut barrier integrity is inextricably linked to a balanced microbial ecosystem, we next investigated whether the structural restoration observed above coincided with a reconfiguration of the gut microbiota composition. As shown by the alpha diversity analysis (Figure 5A–C), chronic stress and GV-971 altered the gut microbial community in different respects. In terms of richness, the Chao1 index (Figure 5A) tended to be lower in the CRS group than in the control group, implying that chronic stress may be accompanied by a reduction in microbial richness. Notably, GV-971 treatment significantly increased the Chao1 index compared with the CRS group (*p* < 0.05), suggesting a partial recovery of stress-associated richness loss. The Shannon index (Figure 5B) was similar between the control and CRS groups, while the GV-971 group showed a tendency toward higher values than both groups. This pattern suggests that GV-971 may modestly enhance overall community diversity, and that its effect may be expressed more through reshaping community structure than through large shifts in diversity itself. The Simpson dominance index (Figure 5C), where lower values reflect less concentration of dominant taxa and greater evenness across the community, revealed that the CRS group showed a downward trend relative to the control group. However, GV-971 treatment led to a further significant decrease, with values notably lower than those of the control group (*p* < 0.01). Taken together, these findings indicate that GV-971 may lessen the over-representation of dominant taxa and help rebalance the microbial community. Beta-diversity analyses in (Figure 5D–F) revealed group-wise differences in overall community structure. In both PCoA plots generated from CLR-Euclidean (Figure 5D) and Bray–Curtis distances (Figure 5E), together with the Bray–Curtis-based NMDS plot (Figure 5F), the CRS group is clearly separated from the control group, consistent with a CRS-induced shift in gut microbial composition. The GV-971 group occupies an intermediate position between CRS and control and appears closer to the control group, suggesting that GV-971 may mitigate, at least in part, the microbial alterations associated with CRS.

Taxonomic profiling at the phylum level showed that Bacteroidota and Bacillota were the two dominant phyla in every group (Figure 5G). In the CRS group, Bacillota decreased slightly, whereas Bacteroidota increased relative to the control group. GV-971 partially normalized these changes, suggesting that it may alleviate the chronic stress-associated disruption of the Bacillota/Bacteroidota ratio. At the species level (Figure 5H), CRS induced a marked shift in gut microbial composition relative to controls, consistent with stress-associated dysbiosis. Specifically, the relative abundances of *Duncaniella dubosii*, *Muribaculum intestinale*, *Bacteroides caecimuris*, *Phocaeicola vulgatus*, *Bacteroides faecium*, *Phocaeicola dorei*, and *Parabacteroides merdae* tended to increase in CRS mice, whereas those of *Paramuribaculum intestinale* and *Muribaculum gordoncarteri* tended to decrease. GV-971 partially reversed these changes, shifting the overall microbial profile toward that of control mice. Several taxa elevated in CRS declined or moved toward baseline after treatment, whereas *Muribaculum gordoncarteri*, *Bacteroides uniformis*, and *Parabacteroides distasonis* increased. These data suggest that GV-971 mitigated CRS-associated disturbances in gut microbial composition and promoted a partial return toward microbial homeostasis.

To identify specific biomarkers associated with GV-971 intervention, we performed LEfSe analysis (Figure 5I). While the CRS group was characterized by the enrichment of *Parabacteroides merdae*, *Parabacteroides johnsonii* and *Acinetobacter radioresistens*, GV-971 treatment significantly expanded the abundance of 22 species, including *Bacteroides uniformis*, *Bifidobacterium pseudolongum*, and *Lactococcus garvieae.* This shift from stress-associated *Parabacteroides* to a more diverse profile of beneficial commensals suggests that GV-971 mitigates CRS-induced dysbiosis, supporting its potential role in modulating the gut–brain axis.

Finally, we predicted the functional potential of the gut microbiome. For a comprehensive overview, we present the 30 pathways showing the most dramatic shifts (Figure 5J). These differential pathways clustered mainly in energy metabolism, inflammatory lipid metabolism, and xenobiotic/drug metabolism. Specifically, GV-971 administration led to a robust enrichment of 13 pathways, notably those involved in xenobiotic biodegradation (e.g., benzoate, naphthalene, and styrene degradation) and energy metabolism, such as the synthesis and degradation of ketone bodies (ko00072). Two cytochrome P450-related pathways (ko00980 and ko00982) and arachidonic acid metabolism (ko00590) also showed increased representation in GV-971-treated mice, suggesting a systemic shift in metabolic and inflammatory signaling. Conversely, GV-971 significantly suppressed phenylpropanoid biosynthesis (ko00940) and cyanoamino acid metabolism (ko00460) in CRS-exposed mice. Other annotated pathways, including those related to glutathione metabolism and NOD-like receptor signaling, did not reach statistical significance. Functional profiling via MetaCyc (Figure 5K) revealed that GV-971 significantly augmented the microbial capacity for amino acid biosynthesis and central carbon metabolism. Specifically, GV-971 treatment led to increased representation of superpathways for L-arginine (PWY-7400), L-lysine (P4-PWY), and L-methionine (PWY-5345) biosynthesis, alongside multiple tricarboxylic acid (TCA) cycle variants (e.g., PWY-6969, REDCITCYC) and fatty-acid oxidation (FAO-PWY). Notably, pathways supporting cofactor synthesis, such as heme b and molybdopterin, were also enriched. In contrast, the CRS group was characterized by a higher abundance of adenine and adenosine salvage (PWY-6609), phosphopantothenate biosynthesis I (PANTO-PWY), and spermidine biosynthesis III (PWY-6834), all of which were attenuated by GV-971. These data indicate that GV-971 reshapes the gut metabolic landscape, shifting it from stress-related salvage processes toward essential anabolic and energetic functions.

### 2.6. GV-971 Modulates Tryptophan Metabolism in Plasma Metabolomic Profiles

**Figure 6:**
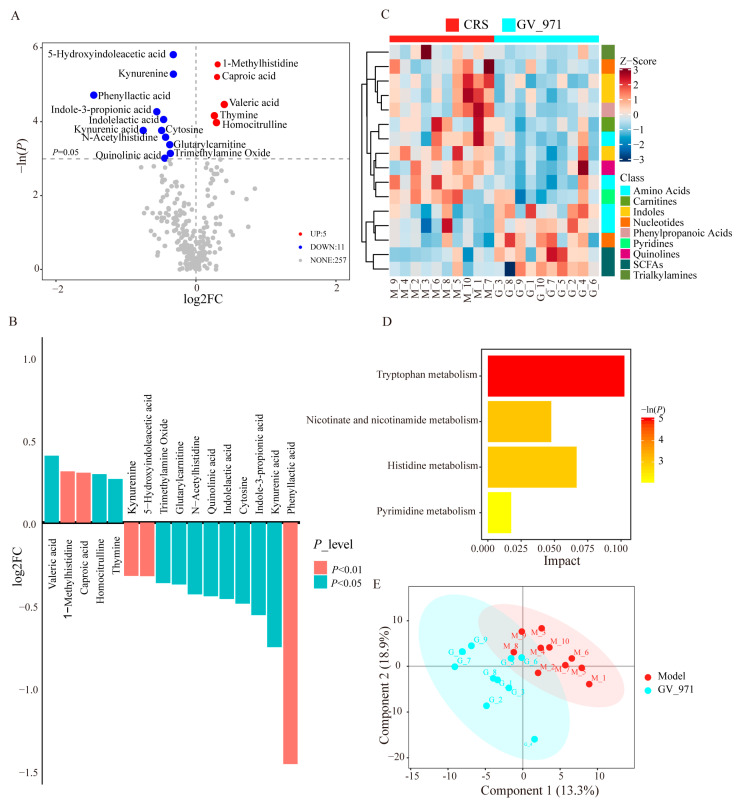
GV-971 treatment modulates tryptophan metabolism in plasma metabolomic profiling. (**A**) Volcano plot of univariate statistics comparing the GV-971 and CRS groups. (**B**) Bar plot illustrating the log_2_ fold change (log_2_FC) and false discovery rate (FDR) of differential metabolic markers. (**C**) Z-score heatmap displaying the relative abundances of differential metabolites. (**D**) Bar plot of pathway enrichment analysis based on the KEGG pathway-associated metabolite sets. (**E**) PLS-DA 2D score plot with labeled sample names. *n* = 10 mice per group.

Given that the gut microbiota functions as a metabolic organ, the taxonomic shifts and functional remodeling observed above prompted us to investigate whether these changes translated into altered levels of key microbial metabolites. Targeted plasma metabolomics was performed to characterize CRS-induced metabolic alterations and their modulation by GV-971. Volcano plot analysis (Figure 6A) identified 16 metabolites that differed significantly between the CRS model and GV-971-treated groups, with 5 metabolites being upregulated and 11 downregulated in response to GV-971. Consistent with this, the bar plot of differential metabolites (Figure 6B) quantitatively illustrates that GV-971 markedly increased short-chain fatty acid-related metabolites, specifically valeric and caproic acids, while significantly reducing a series of metabolites, including key products of the tryptophan pathway such as kynurenine, kynurenic acid, quinolinic acid (QUIN), and 5-hydroxyindoleacetic acid (5-HIAA), as well as phenyllactic acid and indole-3-propionic acid, and trimethylamine oxide (TMAO). These substantially altered metabolites are mainly involved in tryptophan metabolism, nicotinate and nicotinamide metabolism, histidine metabolism, and pyrimidine metabolism, pathways that are closely linked to neuroinflammation and neurotransmitter homeostasis. Together, these data indicate that GV-971 partially reverses the abnormal metabolic signature induced by chronic restraint stress. A heatmap of these discriminant metabolites (Figure 6C) showed clear groupwise clustering. When annotated by chemical class, the altered metabolites encompassed amino acids and their derivatives, indole compounds, nucleotides, and related molecules. GV-971 induced a systematic shift in the relative abundance of these metabolites, yielding a metabolic signature that was clearly separated from that of the CRS group.

Pathway enrichment analysis (Figure 6D) confirmed significant over-representation of tryptophan metabolism, nicotinate and nicotinamide metabolism, and histidine metabolism among the differential metabolites, with tryptophan metabolism showing the strongest enrichment. This pattern highlights tryptophan metabolism as a potential metabolic target underlying the antidepressant-like actions of GV-971. The PLS-DA score plot (Figure 6E) further demonstrates a clear separation between the CRS and GV-971 groups in multivariate space, supporting robust GV-971-induced remodeling of the CRS-altered plasma metabolome. Taken together, these data suggest that GV-971 reshapes metabolic homeostasis in CRS-exposed mice through two major gut–brain axis-related processes. First, GV-971 altered tryptophan metabolism, evidenced by reduced 5-HIAA levels, indicating attenuated 5-HT catabolism, and by lower kynurenine and kynurenic acid levels, suggesting a suppression of the inflammation-associated tryptophan–kynurenine pathway. Second, GV-971 increased valeric and caproic acids and microbial-derived fatty acids with reported anti-inflammatory and neuroprotective potential. Collectively, these metabolic shifts likely contribute to the antidepressant-like effects of GV-971 in CRS-exposed mice.

### 2.7. FMT from GV-971-Treated Donor Mice Attenuates CRS-Induced Depression-like Behavior

**Figure 7:**
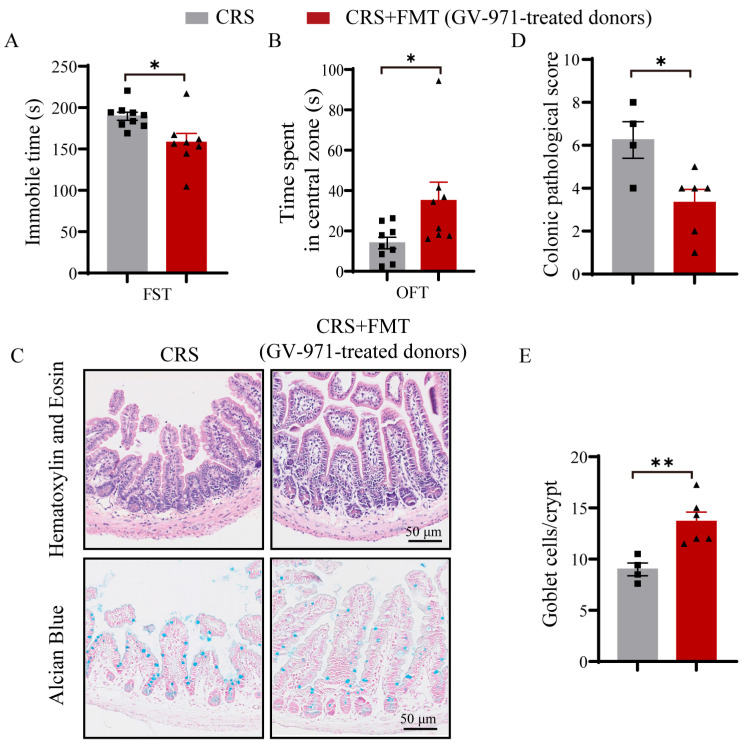
Transplantation of microbiota from GV-971-treated donors improves CRS-induced behavioral deficits and colonic pathology. (**A**) Immobility time in the forced swim test, *n =* 8–9 mice per group. (**B**) Time spent in the center area during the open field test, *n =* 8–9 mice per group. (**C**) Representative H&E and Alcian blue staining of colon sections showing mucosal morphology and mucus production, *n =* 4–6 mice per group. (**D**) Colonic histopathological injury scores. (**E**) Quantification of goblet cells in colonic crypts, *n =* 4–6 mice per group. CRS group (represented by light gray filled squares and black squares), and CRS+FMT group (represented by red filled squares and black upward triangles). Data are presented as mean ± SEM. Statistical significance for comparisons between the CRS group and treatment group was determined using the independent-samples *t*-test. * *p* < 0.05 and ** *p* < 0.01 compared to the CRS group. Scale bars: 50 μm for H&E zoom images and Alcian blue images.

While the concurrent restoration of gut microbiota composition, functional potential, and metabolic profiles following GV-971 treatment suggests a strong association with behavioral improvements, it does not definitively establish causality. To test whether GV-971-induced alterations in the gut microbiota are sufficient to influence stress-related behavior, we performed fecal microbiota transplantation (FMT) from GV-971-treated donor mice into CRS-exposed recipients. After 4 weeks of CRS and FMT, mice were assessed in the open field test (OFT) and forced swim test (FST). In the FST (Figure 7A), mice receiving GV-971-FMT showed a clear reduction in immobility: immobility time fell from 189.59 ± 4.92 s in the CRS group to 157.94 ± 10.87 s (*p* = 0.015), consistent with an attenuation of depression-like behavior. In the OFT (Figure 7B), GV-971-FMT increased the time spent in the central zone from 14.01 ± 2.91 s in the CRS group to 35.07 ± 9.16 s (*p* = 0.036), indicating improved exploratory activity and reduced anxiety-like avoidance. Histological analysis of the colon revealed parallel changes (Figure 7C). Hematoxylin and eosin staining showed that GV-971-FMT preserved mucosal integrity, with more regular crypt architecture and less inflammatory cell infiltration than in the CRS group. The corresponding histological injury score (Figure 7D) decreased from 6.25 ± 0.85 in CRS mice to 3.33 ± 0.61 after GV-971-FMT (*p* = 0.021). Alcian blue staining (Figure 7C) demonstrated stronger mucin-positive signals in colonic crypts in the GV-971-FMT group, and quantification (Figure 7E) confirmed a rise in goblet cell numbers per crypt from 9.00 ± 0.62 in CRS controls to 13.68 ± 0.92 (*p* = 0.0057). Taken together, these findings indicate that microbiota transferred by GV-971-FMT are sufficient to partially reverse CRS-induced behavioral abnormalities and colonic injury, supporting a causal contribution of the gut microbiota to the antidepressant-like actions of GV-971.

## 3. Discussion

GV-971 significantly attenuated CRS-induced depression-like behaviors and HPA-axis hyperactivation, evidenced by reduced plasma corticosterone and restored adrenal morphology. Mechanistically, GV-971 repaired intestinal barrier dysfunction, reversing mucosal injury and goblet cell depletion. Metagenomic analysis showed GV-971 remodeled gut microbiota by suppressing *Parabacteroides* and enriching beneficial taxa like *Bifidobacterium pseudolongum*. Associated with these structural and microbial restorations, GV-971 specifically normalized tryptophan–kynurenine pathway metabolites (including kynurenine, quinolinic acid, and kynurenic acid) and significantly increased fecal short-chain fatty acids (particularly valeric and caproic acids). Collectively, these data indicate GV-971 alleviates depression by restoring gut barrier integrity and rebalancing microbiota–host metabolic crosstalk.

Increasing evidence indicates that AD and major depression share substantial overlap in their pathophysiological underpinnings, particularly at the level of the gut–brain axis, encompassing perturbations in the gut microbial ecosystem, gut barrier disruption with consequent systemic inflammation, and secondary neuroinflammatory responses [17,18]. On this basis, the current work employed a chronic restraint stress (CRS)-induced mouse model of depression to systematically evaluate the antidepressant-like effects of GV-971 and to delineate its multi-target mechanism of action along the “gut microbiota–metabolite–neuroinflammation” axis. GV-971 is a marine-derived oligosaccharide that has been shown to exert robust therapeutic effects in Alzheimer’s disease (AD) by correcting gut dysbiosis, limiting peripheral immune cell infiltration, and attenuating neuroinflammation in the brain [14]. A Phase III clinical trial (NCT02293915) has further demonstrated that GV-971, at a dose of 900 mg/day for up to 36 weeks, is safe and well tolerated in patients with AD [19], providing a solid rationale for the dosing regimen adopted in the present study and supporting its future translational potential. Importantly, the effects of GV-971 on microbiota remodeling and neuroinflammation are not unique; other oligosaccharides have been reported to exert similar functions. For example, neoagaro-oligosaccharides ameliorate CRS-induced depression by increasing brain 5-HT and BDNF and remodeling the gut microbiota [20], and Morinda officinalis oligosaccharide (MOOS), an approved antidepressant in China, mitigate neuroinflammation and depression-like behavior by deactivating the MyD88/PI3K pathway [21]. Therefore, GV-971 shares convergent mechanisms with other oligosaccharides along the gut–brain axis, rather than representing a first or unique discovery.

Impairment of the intestinal barrier and gut microbial dysbiosis are well-recognized peripheral alterations associated with stress-related depression. Histopathological and metagenomic data indicated that GV-971 mitigated CRS-induced colonic crypt disruption and goblet cell depletion, while also decreasing the relative abundance of opportunistic pathogens, including *Parabacteroides*, and increasing the abundance of beneficial taxa such as *Bifidobacterium pseudolongum* and *Butyricimonas*. Members of the genus *Bifidobacterium* have been widely reported to exert psychobiotic effects through modulation of affective behavior and inflammatory tone [22,23,24] whereas *Butyricimonas* is a key SCFA-producing genus in the intestinal ecosystem [25]. These findings suggest that restoration of the intestinal barrier and enrichment of beneficial taxa provide a key microbial basis for the effects of GV-971 on peripheral metabolic regulation.

Tryptophan (Trp) metabolism represents a key interface between the gut microbiota and central nervous system function. In depression, aberrant activation of the kynurenine pathway not only diverts Trp away from 5-hydroxytryptamine (5-HT) synthesis, but also promotes the accumulation of proinflammatory and neuroactive kynurenine pathway metabolites, particularly quinolinic acid (QUIN), thereby exacerbating neuroinflammatory processes in the brain [26,27]. Targeted metabolomics showed that GV-971 significantly reduced peripheral levels of 5-hydroxyindoleacetic acid (5-HIAA), kynurenine (KYN), kynurenic acid (KYNA), and QUIN, while concomitantly increasing hippocampal 5-HT levels. This metabolic profile is consistent with a rebalancing of peripheral Trp catabolism and a restoration of central monoaminergic homeostasis, which may in turn lessen the peripheral neuroactive and inflammatory burden impinging on the brain. In parallel, enrichment of short-chain fatty acid (SCFA)-producing taxa, such as *Butyricimonas*, was accompanied by a significant increase in peripheral SCFA-related metabolites, exemplified by valeric acid. Previous studies have shown that SCFAs not only reinforce intestinal barrier integrity, but also regulate central neuroimmune function after entering the circulation and crossing the blood–brain barrier [28,29]. Taken together, these findings support the view that GV-971 establishes a robust metabolic basis for alleviating chronic stress-induced depressive phenotypes through modulation of Trp metabolism and enhancement of microbiota-derived SCFA signaling.

Excessive activation of the hypothalamic–pituitary–adrenal (HPA) axis, accompanied by elevated circulating corticosterone, represents a central pathophysiological hallmark of major depression. In the present study, CRS exposure induced pronounced depression-like behavior and a marked elevation of plasma corticosterone, whereas GV-971 treatment significantly attenuated this neuroendocrine disturbance. Accumulating evidence indicates that the gut microbiota is a key regulator of HPA-axis development and stress responsiveness [30,31]. Microbial metabolites can signal to the HPA axis via the vagus nerve or by entering the systemic circulation, thereby constraining stress-induced HPA activation [32,33]. These observations suggest that the GV-971-induced reduction in corticosterone and the associated behavioral benefits are likely to arise, at least in part, from its capacity to remodel the gut microbiota and its metabolic outputs.

Regarding the primacy of gut versus brain in the mechanism of GV-971, we propose that intestinal modulation represents an upstream event. GV-971 is a large oligosaccharide molecule that poorly crosses the blood–brain barrier; the vast majority remains in the intestinal tract. In the present study, fecal microbiota transplantation (FMT) from GV-971-treated donor mice into recipient CRS-exposed mice significantly attenuated depression-like behaviors (as shown in Figure 7). This result demonstrates that gut microbiota alterations are sufficient to transfer the antidepressant effects of GV-971, strongly suggesting that gut microbiota remodeling is an upstream event and that suppression of central neuroinflammation may be a downstream consequence of GV-971 action. This interpretation is further bolstered by previous studies demonstrating that GV-971 remodels gut microbiota and suppresses gut-derived peripheral inflammation before exerting central effects [14,16,34]. While FMT experiments support the sufficiency of the microbiota, they do not entirely exclude contributions from other pathways, such as direct systemic metabolic effects or gut–brain neural signaling. Future studies should employ germ-free animal models, defined colonization strategies with specific bacterial strains (e.g., Bifidobacterium pseudolongum), and time-series intervention experiments to more rigorously establish the gut–brain causal chain.

Neuroinflammation driven by aberrant microglial activation is increasingly recognized as a central pathological feature of depression [35,36]. In the present study, immunofluorescence analysis of brain tissue showed that chronic restraint stress (CRS) increased the abundance of Iba1^+^ microglia in the cortex and in key hippocampal subregions, including CA1 and the dentate gyrus (DG), and promoted a shift toward an amoeboid morphology indicative of inflammatory activation. GV-971 markedly attenuated these alterations, as reflected by reduced Iba1 immunoreactivity and partial restoration of a resting-like microglial morphology in these regions. As noted above, gut-derived metabolites function as important signaling mediators in the maintenance of central neuroimmune homeostasis. Previous studies have shown that short-chain fatty acids (SCFAs) of intestinal origin can enter the circulation, reach the brain, and suppress inflammatory polarization and excessive activation of microglia by modulating the central microenvironment [37]. In light of the metabolomic findings, the GV-971-induced increase in peripheral SCFA-related metabolites, including valeric acid, is mechanistically consistent with its inhibitory effect on central microglial activation. These observations suggest that the beneficial effects of GV-971 on depression-like behavior may be partly attributable to remodeling of the gut-derived metabolite profile, thereby attenuating microglia-mediated neuroinflammatory cascades in the brain.

Fluoxetine, as a classic SSRI antidepressant, also exerts modulatory effects on the gut microbiota and neuroinflammation. Recent evidence indicates a bidirectional interaction between antidepressants and the intestinal microbiota [38]. Specifically, fluoxetine has been shown to significantly alter gut microbial composition and diversity, and to reshape the intestinal microenvironment, thereby improving intestinal barrier function and reducing colonic inflammation [39,40]. Moreover, beyond its canonical serotonin reuptake inhibition, fluoxetine possesses direct anti-neuroinflammatory properties. Zhao et al. [41] demonstrated that fluoxetine (10 mg/kg, i.p., 2 weeks) significantly alleviated chronic stress-induced glial activation and suppressed pro-inflammatory cytokines (IL-1β, IFN-γ, TNF-α) in the hippocampus via inhibition of the p38 MAPK pathway. Thus, the observation that fluoxetine reduces neuroinflammation in our study is consistent with the published literature. With respect to the 5-HT findings, the absence of a statistically significant increase in total hippocampal 5-HT in the fluoxetine group warrants explanation. Fluoxetine primarily elevates synaptic cleft 5-HT by inhibiting reuptake, rather than directly increasing total tissue 5-HT content. Total 5-HT levels measured in hippocampal homogenates reflect intracellular and storage pools, which are subject to compensatory mechanisms following chronic treatment, including serotonergic autoregulation via somatodendritic 5-HT_1_A autoreceptors, homeostatic adjustments in 5-HT synthesis and release, and receptor desensitization. Consistent with this, Kulikov et al. [42] reported that chronic SSRI administration (including fluoxetine and paroxetine) may even lead to region-specific 5-HT depletion. Therefore, the lack of a significant increase in total 5-HT in our study is not unexpected and aligns with published pharmacological evidence. We have not performed metagenomic sequencing on fluoxetine-treated fecal samples in the current study, which we acknowledge as a limitation. Future studies should include fluoxetine as a control for microbiota analysis to enable direct comparison of the microbiota-modulating efficacy between GV-971 and conventional SSRIs.

The clinical utility of current antidepressants, especially SSRIs, is limited by delayed onset, incomplete response, and poor tolerability, with adverse gastrointestinal effects among the most frequent treatment-emergent complaints. In addition, many patients fail to achieve remission even after adequate first-line treatment, and residual symptoms commonly persist [4]. These limitations continue to drive the search for antidepressants with improved efficacy and safety profiles. As a marine oligosaccharide targeting the gut–brain axis, GV-971 may help overcome some of the limitations of single-target monoaminergic therapies through coordinated modulation of the microbiota, metabolite signaling, and neuroinflammatory processes. This feature may be particularly relevant for difficult-to-treat depression and for patients in whom gastrointestinal comorbidity complicates standard antidepressant treatment. However, this study has several limitations. The mechanistic and behavioral findings were derived from a single CRS paradigm and should be validated in other depression models, including CUMS and social defeat. Moreover, although FMT points to a transferable microbiota-dependent component, it does not identify the individual bacterial strains or microbial enzyme systems responsible for the effects of GV-971. These questions will be better addressed using germ-free mice, defined colonization strategies, and genetically modified strains, including candidate *Bifidobacterium pseudolongum* isolates. Finally, the relevance of these preclinical observations to human depression remains to be established. Extending this work to clinically relevant comorbidity models, such as AD with depressive phenotypes, may further clarify the therapeutic scope of GV-971.

## 4. Materials and Methods

### 4.1. Experimental Animals

Male C57BL/6J mice (SPF grade, 7–8 weeks old) were sourced from Beijing Vital River Laboratory Animal Technology Co., Ltd., Beijing, China (Production License: SCXK [Beijing] 2021-0011). Animals were group-housed in a barrier-sustained facility under a standard 12 h light/dark cycle, with ambient temperature and relative humidity strictly maintained at 25 ± 2 °C and 50 ± 10%, respectively. Standard chow and water were provided *ad libitum*. Formal experiments commenced following a 3-to-5-day acclimation phase. All experimental operations were approved by the Institutional Animal Care and Use Committee at Shanghai Institute of Materia Medica, IACUC No. 2022-10-GMY-28.

### 4.2. Animal Experimental Design and Chronic Restraint Stress (CRS) Procedure

#### 4.2.1. Optimization and Induction of the CRS Model

Before evaluating the efficacy of GV-971, restraint parameters were pilot-screened in C57BL/6J mice to determine conditions that produced robust depression-like behavior. Immobility time in the forced swim test (FST) was used as the primary endpoint for comparing daily restraint durations of 4 or 8 h across 2-, 4-, and 8-week modeling periods.

Guided by these pilot data, an 8 h daily restraint schedule was adopted for all subsequent studies. Following acclimation, mice were confined within modified 50 mL conical tubes perforated for ventilation. This configuration effectively restricted gross body movement while preserving normal respiration and preventing overt physical injury. The stressor was applied daily from 9:00 to 17:00, during which food and water were restricted for both stressed and control mice. Under these specific parameters, CRS-induced behavioral alterations remained consistently detectable from weeks 4 to 8.

#### 4.2.2. Pharmacodynamic Evaluation of GV-971

Forty-eight male C57BL/6J mice were randomly assigned to three groups (*n =* 16 per group): CRS + vehicle (water), CRS + fluoxetine (10 mg/kg), and CRS + GV-971 (100 mg/kg). GV-971 (batch No. 04240303; Shanghai Green Valley Pharmaceuticals, Shanghai, China) and fluoxetine hydrochloride (Cat. No. F0750; TCI (Shanghai) Development Co., Ltd., Shanghai, China) were freshly dissolved in distilled water to prepare 10 mg/mL and 1 mg/mL solutions, respectively, and vortexed until fully dissolved. Fluoxetine was used as a positive control for antidepressant efficacy.

For 7 weeks, fluoxetine and GV-971 were administered once daily by intragastric (i.g.) gavage at 10 mL/kg, and CRS + vehicle mice received water by the same route and volume. Behavioral testing was performed between weeks 5 and 7. Behavioral assessments included the elevated plus maze, tail suspension test, and forced swim test, with at least 48 h between tests. CRS exposure was continued every day, including during these intervals. At the end of week 7, all animals were euthanized, and brain and peripheral tissues were collected for biochemical analyses.

#### 4.2.3. Fecal Microbiota Transplantation (FMT) Validation Study

To test whether the behavioral effects of GV-971 depended on the gut microbiota, 20 male C57BL/6J mice were randomly assigned to a CRS control group and an FMT group (*n =* 10 per group). Following [43], mice in the FMT group were rendered pseudo-germ-free with a 3-day microbiota-depletion regimen consisting of twice-daily oral gavage of a broad-spectrum antibiotic cocktail: cefalexin (100 mg/kg; C9600, Solarbio, Beijing, China), oxytetracycline hydrochloride (300 mg/kg; O8060, Solarbio), and erythromycin (300 mg/kg; E8100, Solarbio, Beijing, China).

Donor fecal suspensions were prepared freshly each day under anaerobic conditions in an anaerobic workstation (MAWORDE, YY-XXL PLUS, Shanghai, China). Briefly, 80 mg of freshly voided fecal pellets from GV-971-treated donor mice were homogenized in 2 mL of sterile PBS containing 0.05% DL-cysteine HCl (cat. no. 01002914, Adamas, Shanghai, China) to obtain a 4% (*w*/*v*) suspension. The homogenate was passed through a sterile 70 μm filter, and the filtrate was administered immediately after preparation.

After antibiotic pretreatment, both groups were subjected to the 7-week CRS protocol. During CRS, the FMT group received 0.2 mL/day of the donor fecal suspension by oral gavage, whereas CRS controls were given an equal volume of vehicle. At the end of the 7-week protocol, mice underwent behavioral testing as described above.

### 4.3. Behavioral Tests

#### 4.3.1. Open Field Test (OFT)

Mice were gently placed in the center of a square open-field arena (40 × 40 × 40 cm^3^) and allowed to explore freely for 10 min. Locomotor activity was recorded and analyzed using an automated video-tracking system (EthoVision XT 17, Noldus Information Technology, Wageningen, The Netherlands). Time spent in the center zone was quantified. The arena was wiped with 10% ethanol between trials to eliminate olfactory cues.

#### 4.3.2. Elevated Plus Maze (EPM)

For the EPM test, mice were placed in the central platform of the maze facing an open arm and allowed to explore for 5 min. Activity was recorded using EthoVision XT 17 (Noldus Information Technology, Wageningen, The Netherlands), and the distance traveled in the open arms was measured as an index of anxiety-like behavior. The apparatus was cleaned with 10% ethanol between animals.

#### 4.3.3. Forced Swim Test (FST)

Mice were individually placed in a transparent cylindrical tank (diameter: 16 cm) filled with water (25 ± 1 °C; depth 20 cm), preventing the tail and hind paws from touching the bottom. Behavior was recorded for 6 min, and immobility time during the last 4 min was quantified using EthoVision XT 17 (Noldus Information Technology, Wageningen, The Netherlands).

#### 4.3.4. Tail Suspension Test (TST)

For the TST, the tail of each mouse was fixed to a suspension bar with adhesive tape approximately 1 cm from the tip, leaving the animal hanging head-down 60 cm above the surface. Sessions lasted 6 min, and immobility time during the last 4 min was scored with EthoVision XT 17 (Noldus Information Technology, Wageningen, The Netherlands). Immobility was defined as the absence of active escape-directed movements, with the body and limbs remaining motionless.

### 4.4. Intestinal Permeability Assay (FITC-Dextran)

Mice were fasted for 12 h with free access to water before the assay. FITC-dextran (4 kDa; FD4-1G, Sigma-Aldrich (Merck KGaA, Shanghai, China) was administered by oral gavage at 600 mg/kg. Two hours later, approximately 120 μL of blood was collected from the retro-orbital venous plexus and centrifuged at 12,000× *g* for 3 min at 4 °C. Plasma was diluted 1:1 with PBS (pH: 7.4) in black 96-well plates. Fluorescence was measured using a multimode plate reader (EnVision, PerkinElmer, Waltham, MA, USA), and FITC-dextran concentrations were calculated from a standard curve.

### 4.5. Sample Collection and Processing

Upon completion of behavioral testing, animals were anesthetized with Zoletil 50 (25 mg/kg, i.p.) and transcardially perfused with ice-cold physiological saline for 5 min to clear circulating blood. After hepatic blanching and decapitation, brains were swiftly harvested. Guided by a mouse brain stereotaxic atlas [44], the prefrontal cortex and hippocampus were carefully microdissected and snap-frozen for the neurotransmitter assays. In parallel, colonic tissues were isolated and fixed for histological analysis.

### 4.6. Hematoxylin and Eosin Staining

Distal colonic segments were fixed in 4% paraformaldehyde for 24 h, processed, embedded in paraffin, and sectioned at 3 μm. Sections were placed on glass slides, deparaffinized in xylene substitute (Cat. No. BA7002C, Baso, Zhuhai, China; three changes, 5 min each), and rehydrated through graded ethanol (100%, 90%, 80%, and 70%) before immersion in distilled water for 2 min. Hematoxylin–eosin staining was performed using a commercial kit (Cat. No. C0105S, Beyotime, Shanghai, China). Sections were stained with hematoxylin for 6 min, rinsed in running tap water, briefly differentiated for 5 s, and blued in running tap water for 10 min. After a brief rinse in distilled water, sections were counterstained with eosin for 3 s, rapidly dehydrated through 70%, 80%, 90%, and 100% ethanol (for approximately 10 s each), cleared in xylene substitute (three changes, 20 s each), and mounted with a slide mounting medium (Cat. No. 110110003, BKMAMLAB, Changde, China). Slides were digitized using a PhenoImager tissue imaging system (Akoya Biosciences, Marlborough, MA, USA). Histological damage was evaluated using a four-parameter scoring system (0–3 per parameter; maximum score: 12) that considered epithelial injury and goblet cell depletion, leukocyte infiltration in the lamina propria, the extent of tissue involvement, and severe inflammatory features, including crypt abscesses and mucosal edema [45].

### 4.7. Alcian Blue–Nuclear Fast Red Staining and Goblet Cell Quantification

For goblet cell evaluation, adjacent 3 μm colonic sections were deparaffinized in xylene substitute and rehydrated as described above. Alcian blue–nuclear fast red staining was performed using a commercial kit (Cat. No. C0155M, Beyotime, Shanghai, China). Sections were incubated in 1% Alcian blue solution (pH: 2.5) for 30 min at room temperature, rinsed in running tap water for 2 min, and washed twice in deionized water for 2 min each. Nuclei were then counterstained with 0.1% nuclear fast red for 5 min at room temperature, followed by rinsing in running tap water for 2 min and two further washes in deionized water for 2 min each. Slides were subsequently dehydrated, cleared, and mounted with a slide mounting medium, and then scanned using a PhenoImager tissue imaging system. Goblet cell numbers were quantified by direct counting, and the mean number of goblet cells per crypt was calculated.

### 4.8. Immunofluorescence Staining of Brain Sections

Fixed whole brains were cryoprotected in 10%, 20%, and 30% sucrose solutions until they sank, embedded in Tissue-Tek^®^ O.C.T. Compound (Cat. No. 4583, Sakura Finetek, Torrance, CA, USA), and cut into 30 μm coronal sections on a cryostat (CM1950, Leica, Wetzlar, Germany). Sections containing the dorsal hippocampus (Bregma AP, −1.8 to −2.2 mm) were collected, mounted onto adhesive slides, air-dried at 37 °C, and post-fixed in 4% paraformaldehyde for 10 min at room temperature. After washing with PBS (three times, 5 min each), sections were permeabilized with PBS containing 0.3% Triton X-100 (Cat. No. 85111, Thermo Fisher Scientific, Waltham, MA, USA) for 10 min at room temperature and blocked with 10% goat serum (Cat. No. C0265, Beyotime, Shanghai, China) for 2 h. Sections were then incubated with chicken anti-IBA1 antibody (1:800, Cat. No. 234009, Synaptic Systems, Göttingen, Germany) at 4 °C for 48 h in a humidified chamber. After PBS washes, sections were incubated with Alexa Fluor™ 647-conjugated goat anti-chicken IgY (H + L) secondary antibody (1:500; Cat. No. A-21449; Thermo Fisher Scientific, Waltham, MA, USA) for 2 h at room temperature in the dark. Following further washes, sections were coverslipped with antifade mounting medium containing DAPI (Cat. No. P0131, Beyotime, Shanghai, China). Images of the hippocampus and cortex were acquired using a laser scanning confocal microscope (FV3000, Olympus, Tokyo, Japan) under identical imaging settings. Quantitative analysis was performed in ImageJ 1.54g (NIH, Bethesda, MD, USA). Microglial activation status and IBA1 expression levels were determined by calculating the mean fluorescence intensity (MFI) of IBA1 within individual imaging fields. Meanwhile, the fraction of Iba1-immunopositive cells relative to total DAPI-labeled nuclei was quantified to further characterize microglial population changes.

### 4.9. Quantification of Neurotransmitters and Corticosterone

#### 4.9.1. Brain Tissue Sample Preparation

Brain tissue samples (approximately 20 mg) was accurately weighed and homogenized in 100 μL of ultrapure water. Then 300 μL of precooled methanol was added, and the mixture was vortexed, followed by ultrasonic extraction at 0 °C for 10 min to precipitate the protein. Next, samples were centrifuged at 14,000× *g* for 15 min at 4 °C and 100 μL of the supernatant was transferred to an autosampler vial and kept at 4 °C before analysis.

#### 4.9.2. Plasma Sample Preparation

Plasma samples were thawed on ice, and 20 μL of plasma was mixed with 80 μL of precooled acetonitrile for protein precipitation. The mixture was vortexed and subjected to ultrasonication at 4 °C for 10 min, followed by centrifugation at 14,000× *g* at 4 °C for 20 min. Then 40 μL of the supernatant was transferred to an autosampler vial for analysis.

#### 4.9.3. UPLC-MS/MS Analysis

Norepinephrine (Cat. No. HY-13715, MCE, Shanghai, China), serotonin (Cat. No. HY-B1473, MCE, Shanghai, China), and corticosterone (Cat. No. C0388, TCI(Shanghai) Development Co., Ltd., Shanghai, China) were quantified in multiple reaction monitoring (MRM) mode.

Prepared samples were analyzed on a Nexera LC-40 UPLC system (Shimadzu, Kyoto, Japan) coupled to a Triple Quad™ 7500 system (AB Sciex, Framingham, MA, USA). Chromatographic separation was performed on an HSS T3 column (particle size: 1.8 μm; 2.1 mm (i.d.) × 100 mm (length), Waters). The column temperature was maintained at 40 °C and mobile phases A and B were water and acetonitrile (ACN) containing 0.1% formic acid, respectively. The flow rate was 0.2 mL/min and the gradient was set as follows: 0–2 min, 5% B; 2–7 min, 5–85% B; 7–9 min, 85–90% B; 9–9.1 min, 90–5% B; 9.1–12 min, 5% B. The volume of injection was 5 μL. Data were acquired in MRM mode with positive ion polarity (ESI+). The source parameters were set as follows: spray voltage, 5.5 kV; source temperature, 480 °C; nebulizing gas, 50 psi; auxiliary heating gas, 50 psi; curtain gas, 40 psi. The selected Q1/Q3 pairs for each component are shown in Table 1.

### 4.10. Fecal Metagenomic Sequencing and Analysis

Fresh fecal pellets were collected from mice under sterile conditions, snap-frozen in liquid nitrogen, and stored at −80 °C until analysis. DNA extraction and sequencing were outsourced to Majorbio Pharma & Biotech Co., Ltd. (Shanghai, China). After homogenization using a FastPrep-24 5G instrument, total DNA was extracted with the FastPure Stool DNA Isolation Kit. DNA samples that passed quality control were used to construct sequencing libraries with the NEXTFLEX Rapid DNA-Seq kit and subjected to paired-end metagenomic sequencing on an Illumina NovaSeq™ X Plus platform.

Fecal metagenomic sequencing data were processed via the nf-core/taxprofiler pipeline (v1.1.8). Raw reads were assessed for quality using FastQC, and adapters and low-quality bases were removed with fastp. Host-derived sequences were depleted by mapping against the mouse reference genome (GRCm39) using Bowtie2. Taxonomic classification and species-level abundance re-estimation were performed utilizing Kraken2 and Bracken, respectively. Data were subsequently imported into R (v4.4.1) via the phyloseq package. For alpha diversity, abundance tables were rarefied to an even sequencing depth; Chao1, Shannon, and Simpson indices were compared using either Wilcoxon rank-sum or Kruskal–Wallis tests. Beta diversity (Bray–Curtis distances) was visualized via principal coordinate analysis (PCoA) and non-metric multidimensional scaling (NMDS), with community-level structural differences statistically evaluated by PERMANOVA (adonis2; vegan). Differentially abundant taxonomic biomarkers were identified using LEfSe (microbiome Marker package; LDA score > 2, *p* < 0.05). Functional profiling was executed utilizing HUMAnN3 to quantify gene families, KEGG orthologs (KOs), and pathways. Following relative abundance normalization, functional shifts across groups were evaluated. Where applicable, all *p*-values across diversity and functional analyses were adjusted for multiple testing using the Benjamini–Hochberg method.

### 4.11. Targeted Metabolomics and Analysis

Targeted metabolomics profiling of plasma samples was conducted by Metabo-Profile Biotechnology (Shanghai, China) using the Q500 panel. Sample preparation followed the manufacturer’s standard protocol: Briefly, 50 μL of plasma was mixed with 300 μL of ice-cold methanol containing internal standards for protein precipitation. The mixture was homogenized, incubated at −20 °C, and centrifuged at 18,000× *g* for 20 min at 4 °C. The supernatant was analyzed using an Ultra-Performance Liquid Chromatography–Tandem Mass Spectrometry (UPLC-MS/MS) system, comprising either an ACQUITY UPLC-Xevo TQ-S (Waters, Milford, MA, USA) or a SCIEX Triple Quad™ 6500+ QTRAP (AB Sciex, Framingham, MA, USA). Chromatographic separation was achieved on an ACQUITY UPLC BEH Amide column maintained at 40 °C, optimized for polar metabolite retention. Quality control (QC) samples, prepared by pooling equal volumes of all study samples, were injected periodically throughout the sequence to monitor system stability and data reproducibility. The raw data files generated by UPLC-MS/MS were processed using the TMBQ software (v1.0, Metabo-Profile, Shanghai, China) to perform peak integration, calibration, and quantitation for each metabolite. The self-developed platform iMAP (v1.0, Metabo-Profile, Shanghai, China) was used for statistical analyses.

### 4.12. Statistical Methods

Statistical analyses were performed using SPSS 26.0 (IBM Corp., Armonk, NY, USA) and visualized with GraphPad Prism 9.0. Data are presented as mean ± SEM. After confirming normality via the Shapiro–Wilk test, one-way ANOVA followed by Tukey’s post hoc test was employed to evaluate inter-group differences in the model optimization experiments (control, CRS 4 h, and CRS 8 h). For treatment experiments, independent-samples *t*-tests were employed for comparisons between two groups. Statistical significance was defined as *p* < 0.05.

## 5. Conclusions

In summary, the present study demonstrates that GV-971 shows significant antidepressant-like effects in mice exposed to chronic stress. These effects are associated with coordinated modulation of the gut–brain axis, including remodeling of the gut microbiota, normalization of tryptophan and short-chain fatty acid metabolism, restoration of intestinal barrier integrity, and attenuation of microglia-related neuroinflammation. Collectively, the findings support gut–brain axis-directed pharmacological strategies for depression and provide a preclinical basis for extending the therapeutic application of GV-971 beyond Alzheimer’s disease.

## Figures and Tables

**Table 1:** MRM parameters for the target compounds.

Component	Q1 *m*/*z*	Q3 *m*/*z*	EP (V)	CE (V)	CXP (V)
Norepinephrine	170.1	152.0	10	13	10
Serotonin	177.1	160.0	10	20	8
Corticosterone	347.3	311.2	10	23	17

## Data Availability

Data will be made available on request.

## Abbreviations

The following abbreviations are used in this manuscript:

AD	Alzheimer’s disease
ACN	Acetonitrile
CORT	Corticosterone
CRS	Chronic restraint stress
DG	Dentate gyrus
EPM	Elevated plus maze
ESI+	Electrospray ionization positive
FAO-PWY	Fatty-acid-oxidation superpathway
FDR	False discovery rate
FITC	Fluorescein isothiocyanate
FMT	Fecal microbiota transplantation
FST	Forced swim test
GV-971	Sodium oligomannate
H&E	Hematoxylin and eosin
HPA	Hypothalamic–pituitary–adrenal
IBA1	Ionized calcium-binding adapter molecule 1
KO	KEGG ortholog
KYNA	Kynurenic acid
KYN	Kynurenine
LDA	Linear discriminant analysis
LEfSe	Linear discriminant analysis effect size
MDD	Major depressive disorder
MFI	Mean fluorescence intensity
MRM	Multiple reaction monitoring
NE	Norepinephrine
NMDS	Non-metric multidimensional scaling
OFT	Open field test
PCoA	Principal coordinate analysis
PERMANOVA	Permutational multivariate analysis of variance
PLS-DA	Partial least squares discriminant analysis
QUIN	Quinolinic acid
SCFA	Short-chain fatty acid
SEM	Standard error of the mean
SSRI	Selective serotonin reuptake inhibitor
TCA	Tricarboxylic acid
TMAO	Trimethylamine oxide
TST	Tail suspension test
Trp	Tryptophan
5-HIAA	5-hydroxyindoleacetic acid
5-HT	5-hydroxytryptamine/serotonin
ZF	Zona fasciculata
